# Isorhamnetin Influences the Viability, Superoxide Production and Interleukin-8 Biosynthesis of Human Colorectal Adenocarcinoma HT-29 Cells In Vitro

**DOI:** 10.3390/life13091921

**Published:** 2023-09-15

**Authors:** Hana Greifová, Katarína Tokárová, Tomáš Jambor, Nikola Štefunková, Ivana Speváková, Rudolf Dupák, Olha Balytska, Denis Bažány, Marcela Capcarová, Norbert Lukáč

**Affiliations:** 1 Institute of Applied Biology, Faculty of Biotechnology and Food Sciences, Slovak University of Agriculture in Nitra, Tr. A. Hlinku 2, 949 76 Nitra, Slovakia; hana.greifova@uniag.sk (H.G.);; 2 AgroBioTech Research Centre, Slovak University of Agriculture in Nitra, Tr. A. Hlinku 2, 949 76 Nitra, Slovakia

**Keywords:** isorhamnetin, HT-29 cells, colorectal cancer, proliferation, reactive oxygen species, interleukin 8

## Abstract

Isorhamnetin has gained research interest for its anti-inflammatory, anti-proliferative and chemoprotective properties. In this study, human colon adenocarcinoma cells were cultured in the presence or absence of different isorhamnetin concentrations (5–150 μM) for 24 h or 48 h of cultivation to explore the impact on several parameters of viability/proliferation (mitochondrial function using an MTT test, metabolic activity, cell membrane integrity and lysosomal activity using a triple test). The intracellular generation of superoxide radicals using an NBT test and ELISA analysis was performed to observe the biosynthesis of interleukin 8 (IL-8) in cells stimulated with zymosan, as well as in basal conditions. The antiproliferative activity of isorhamnetin was demonstrated by significantly reduced values of mitochondrial and metabolic activity, integrity of cell membranes and lysosomal activity. Its high prooxidant potential was reflected by the significantly elevated generation of superoxides even in cells with low viability status. The anti-inflammatory effect of isorhamnetin was evident due to decreased IL-8 production, and the most significant decline in IL-8 concentration was observed after 24 h treatment in cells with induced inflammation. We demonstrated that isorhamnetin can suppress the proliferation of HT-29 cells, and this effect was correlated with pro-oxidative and anti-inflammatory activity of isorhamnetin.

## 1. Introduction

Colon cancer represents almost 10% of all tumors, and is the third most diagnosed cancer in men and the second in women in modern countries, with around 1 million new cases identified each year. About 5–10% of instances are genetically based, whereas more than 70% are influenced by nutrition and lifestyle. Crohn’s disease and ulcerative colitis, both of which cause chronic digestive system inflammation and raise the risk of colorectal cancer as well [1]. Additionally, the relatively high reactive oxygen species (ROS) concentration in the tissues of patients with inflammatory diseases of the digestive system, causing irreversible damage to the gastrointestinal structure and function due to ROS-responsive systems, is associated with the development of colon cancer [2]. The main causes of colorectal cancer are thought to be high-fat diets, obesity, alcohol consumption and chronic inflammation of the digestive system. The primary scientific issues of current oncology research are, therefore, the mechanisms of colon cancer’s origin, development, therapeutic intervention and prevention [3]. The use of natural food supplements and dietary changes appears to be an effective and significant method for preventing colorectal cancer. A diet rich in vegetables, fruits, grains and seeds has been associated by epidemiological research with a lower incidence of colon cancer, and dietary constituents like polyphenols have been shown to lessen the risk of colon cancer in animal studies [4].

Several chemokines, including interleukin 8 (IL-8), a member of the chemokine subfamily (CXC) that stimulates cell migration, are released by colorectal carcinomas and the cell lines generated from them. It has been demonstrated that IL-8, a pro-inflammatory cytokine, is a potent chemoattractant for leucocytes and natural killer cells—key components of anticancer immunity [5]. According to recent studies, IL-8 causes the migration of different cell types, with cell migration serving as an important mechanism for the development of metastases with the induction of angiogenesis [6,7,8]. Its expression was linked to angiogenesis, tumor growth and the possibility of metastasis in several human and animal carcinomas [8,9,10]. In this context, IL-8 production is an important marker for tracking carcinogenesis, because angiogenesis plays a key role in the growth of tumors and metastasis; therefore, it represents a promising target for cancer therapy [11]. The hypothesis that IL-8 expression in colon cancer cells corresponds with their capacity to metastasize, and that IL-8 can act as an autocrine/paracrine growth factor during the development and metastasis of colon cancer, is being supported by a growing body of research [12,13].

Despite the fact that there are many different options for colon cancer therapy, the use of natural sources with bioactive compounds for cancer treatment and prevention has significantly increased over the past several years. The leaves, flowers and fruits of *Hippophae rhamnoides* L., *Ginkgo biloba* L. and other plants that have been used as potent ethnopharmaceuticals for ages naturally contain isorhamnetin, a flavonoid component with many beneficial effects [14]. Isorhamnetin-rich extracts have been linked to a number of biological activities, including anti-inflammatory and anticancer effects. Studies have also revealed its antioxidant, antibacterial, anti-obesity, anti-diabetic and hepatic anti-steatotic properties [15]. Isorhamnetin and certain isorhamnetin derivatives, such as isorhamnetin 3-O-d-glucopyranoside, isorhamnetin 3-O-neohesperidoside and isorhamnetin 3-O-rutinoside, are found naturally in a variety of plant materials [16]; therefore, many fruits and vegetables are significant sources of isorhamnetin, in addition to the fact that it is metabolically derived from quercetin [17]. Isorhamnetin has a bioavailability that is several times higher than quercetin, since the metabolism and absorption of methylated flavonoids are significantly improved when compared to their unmethylated parent molecules. These facts suggest that the intrinsic biological activities of isorhamnetin represent a promising target of research interest [18].

Isorhamnetin has been shown to be a potent anticancer agent in in vivo experiments using rodents, particularly mice, and in vitro research using certain cancer cell lines. It has been demonstrated to have an effect in suppressing cancer cell proliferation via the PI3K-Akt-mTOR pathway [19]. Moreover, it has been demonstrated that mitotic block can be enhanced in cancer cells by improving the G2/M arrest caused by cisplatin and carboplatin and to prevent cancer cells from multiplication by halting the G2/M phase of mitosis in general [20,21]. It appears that the ability of isorhamnetin to arrest the cell cycle depends on the specific cancer cell line because, in other cancer cells, it induces S-phase arrest [22,23]. In addition, the death receptor (DR)-dependent extrinsic and/or mitochondria-dependent intrinsic pathways, which are typical apoptosis-inducing pathways, have been linked to isorhamnetin’s anticancer effect [20,24,25,26,27,28,29]. It has also been noted that various key cellular signaling pathways were compromised as a result of isorhamnetin’s anticancer effect [21,22].

In order to carry out normal biological processes like differentiation and development, and to sustain an elevated metabolic rate, cancer cells often require high concentrations of ROS. Additionally, ROS in the necessary amount aid in tumor development, metastasis and angiogenesis. The quantity of ROS, however, exceeds the threshold, causing oxidative damage to the cells, particularly in cancer cells. The increased mitochondrial dysfunction makes the cancer cells more vulnerable to oxidative stress than normal cells. Therefore, a minor rise in ROS levels caused by external stimuli can cause cancer cell death through a variety of death pathways [30]. This mechanism has led to the development of pro-oxidant therapy, which uses agents to cause oxidation-induced cell death, as a potent method of cancer treatment [31]. The pro-oxidant effect of certain bioactive substances with anti-cancer potential, such as damaging cellular antioxidant systems or ROS overproduction and accumulation, are thought to cause apoptosis of cancer cells, according to a growing body of research evidence [32,33]. Considering this, isorhamnetin showed a potent cytotoxic effect on cancer cells via an ROS-dependent apoptotic mechanism [23,34].

In this study, we investigated the in vitro dose- and time-dependent antiproliferative potential of isorhamnetin on HT-29 cells, i.e., human colon adenocarcinoma isolated from a primary tumor, to confirm or deny the cytotoxicity of isorhamnetin (5–150 μM) associated with the antioxidant/prooxidant and anti-inflammatory activity of isorhamnetin. This was carried out as it is proven that carcinogenesis is closely associated with inflammatory processes and with changes in intracellular redox balance. Our hypothesis assumes that the anti-cancer effect of isorhamnetin is related to its ability to interfere with the biosynthesis of interleukin 8, which is a cytokine released from cancer cells that regulates the processes of proliferation, angiogenesis and migration of cancer cells, and is related to the potential of isorhamnetin to affect the oxidative status of cancer cells as well.

## 2. Materials and Methods

### 2.1. Cell Culture and Treatment

Human colon cancer cell line HT-29 was procured from American Type Culture Collection (ATCC HTB-38; Manassas, VA, USA). Protocols which had already been established and approved were used to cultivate the cells. The ATCC batch of HT-29 cells was first cultivated for a minimum of five passages before being split and stored in liquid nitrogen. The HT-29 cell line was maintained in typical T75 cell culture flasks (Corning, NY, USA) at 37 °C in a humidified incubator with 5% CO_2_ and grown in Dulbecco’s modified Eagle’s media (DMEM; Sigma Aldrich, St. Louis, MO, USA) supplemented with 10% fetal bovine serum (FBS; Merck, Berlin, Germany) and 1% penicillin/streptomycin (Invitrogen, Carlsbad, CA, USA). Cells that reached 80% confluent monolayer were subcultured using 0.05% trypsin-ethylenediaminetetraacetic acid solution (Sigma Aldrich, St. Louis, MO, USA) and the cell growth medium was replaced every two days. Cells were seeded onto 96-well plates (1 × 10^5^ cells/well) and allowed to adhere for 24 h prior to treatment. Cells were treated for 24 or 48 h with different concentrations of isorhamnetin (Sigma Aldrich, St. Louis, MO, USA) (experimental groups with 5–150 μM) or without it (control group). Before each experiment, isorhamnetin was dissolved in dimethyl sulfoxide (DMSO; Sigma Aldrich, St. Louis, MO, USA) and diluted with the medium to the final experimental concentrations. The same concentration of DMSO (0.1%) as that used in the experimental group exposed to the highest dose of isorhamnetin was used to treat the controls (C+ group). HT-29 cells were cultured in the presence of zymosan (Sigma Aldrich, St. Louis, MO, USA) at a concentration of 10 μg/mL, along with escalating dosages of isorhamnetin for 24 or 48 h, in order to quantify interleukin 8 in the experimental groups with induced inflammation.

### 2.2. Mitochondrial Activity Assay

The MTT (3-4,5-dimetyltiazol-2-yl)-2,5-diphenyltetrazolium bromide) test was used to analyze the mitochondrial activity of HT-29 cells exposed to isorhamnetin. This assay measures the transformation of a yellow tetrazolium salt into an insoluble blue formazan in living, intact cells [35]. After 24 and 48 h of isorhamnetin treatment, cells were exposed to tetrazolium salt (Sigma-Aldrich, St. Louis, MO, USA) dissolved in culture media for 1 h in a CO_2_ incubator. Subsequently, the supernatants were removed from the wells, and dimethyl sulfoxide was added to dissolve the formazan crystals that had formed in the cells. Using an ELISA reader (Multiscan FC, ThermoFisher Scientific, Vantaa, FL, USA), the samples’ absorbance levels were evaluated at wavelengths of 570 nm against 620 nm. To collect the experimental and control cell samples, four different, independent experiments were run. All statistics are presented as percentages of control (C+, isorhamnetin untreated cells).

### 2.3. Triple Viability/Proliferation Assay

Three different cellular processes after isorhamnetin treatment were observed using different indicator dyes to determine cell viability and cell proliferation: the metabolic activity of cells using resazurin (alamarBlue; Sigma-Aldrich, USA); the integrity of the cells’ plasma membranes using CFDA-AM (5-carboxyfluorescein diacetate acetoxymethyl ester; Sigma-Aldrich, USA); and the integrity of the lysosomes using neutral red (NR; Sigma-Aldrich, USA).

The alamarBlue test is a well-established cell viability indicator that uses the natural reducing power of live cells to convert resazurin to the fluorescent molecule, resorufin. As resazurin is converted to resorufin by mitochondrial and other enzymes like diaphorases, the quantity of intact cells can be assessed based on this non-toxic, cell-permeable redox indicator. Resazurin is converted into the brightly fluorescent compound resorufin of red color once it enters cells. Resazurin is continually metabolized by living cells to resorufin, raising the fluorescence of the culture media.

Another fluorescent dye that shows the integrity of the treated cells’ plasma membrane is CFDA-AM. This compound represents a nontoxic esterase substrate that can be converted from a membrane-permeable, nonpolar, nonfluorescent substance to polar, fluorescent carboxyfluorescein by nonspecific esterases of viable cells. Since only an intact cell plasma membrane can preserve the cytoplasmic environment required to allow for esterase activity, this conversion suggests the rate of integrity of the plasma membrane.

Staining with neutral red constitutes a colorimetric assay. After passing through cell membranes by nonionic passive diffusion, this weakly cationic dye concentrates in the lysosomes. Utilizing an acidified ethanol as a lysis buffer to release the dye from the living cells, the absorbance of samples can then be determined spectrophotometrically.

The use of these 3 different dyes simultaneously to provide a summary of the cytotoxicity/cytoprotectivity of treatments to the cells in 96-well plates was initially described by Schirmer et al. [36]. This technique allows for the interference-free measurement of three cell proliferation parameters at the same time on the same batch of cells. With a few minor modifications, this technique was followed in this study. Briefly, a solution of almarBlue and CFDA-AM in MEM medium (Minimum essential medium eagle; SigmaAldrich, St. Louis, MO, USA) was first added to the cells seeded in the 96-well plate after 24 or 48 h of isorhamnetin treatment. In a humidified incubator with 5% CO_2_, the cells were cultivated for one hour at 37 °C. Subsequent measurements were performed using a Glomax Multi Combined Spectro-Fluoro Luminometer (Promega Corp., Madison, WI, USA) at wavelengths of 485 nm/530 nm for CFDA-AM and 530–560 nm/590 nm, respectively, for alamarBlue. The cells adhered in wells were next rinsed twice with PBS (phosphate-buffered saline; Sigma-Aldrich, St. Louis, MO, USA) before being treated with neutral red dye in MEM medium for an additional one hour. Following an incubation period, the cells were again washed twice with PBS and exposed for another 30 min to the lysis buffer (acidified ethanol). The analysis itself was carried out by means of a Glomax Multi Combined Spectro-Fluoro-Luminometer (Promega Corp., USA) at specific wavelengths. (525/660–720 nm). Data were collected from four separate experiments with cells of different passages and were expressed as percentages of the control, which represented 100%.

### 2.4. Assessment of Intracellular Superoxide Radical Generation

The NBT test, or nitroblue tetrazolium test, measures and quantifies intracellularly generated superoxide radicals. Nitroblue tetrazolium chloride becomes reduced by free oxygen radicals, forming a blue-black compound and formazan deposits within cells, and this reaction can be monitored spectrophotometrically. The NBT reaction reflects the ROS-generating activity in the cytoplasm of cells. Moreover, the results of NBT staining strongly correlate with the ROS levels assessed by chemiluminescence.

HT-29 cells were seeded on 96-well plates, and NBT salt (2,2′-bis(4-nitrophenyl)-5,5′-diphenyl3,3′-(3,3′-dimethoxy-4,4′diphenylene) ditetrazolium chloride; Sigma-Aldrich, St. Louis, MO, USA) was added into the wells after it had been dissolved in growth media containing 1.5% DMSO (dimethyl sulfoxide; Sigma-Aldrich, St. Louis, MO, USA). The cells were washed three times with PBS after being incubated for 3 h (37 °C, 95% air atmosphere, 5% CO_2_) to avoid measuring extracellular superoxide in the samples. Finally, formazan deposits in the cells were released by dissolving them in DMSO after the cells were permeabilized by adding 2 M KOH (potassium hydroxide; Centralchem, Bratislava, Slovakia). The Multiscan FC microplate reader (Multiscan FC, ThermoFisher Scientific, Vantaa, FL, USA) was used to measure the optical density of the samples at a wavelength of 620 nm against a reference wavelength of 570 nm. Data were collected from four separate experiments with cells of different passages and were expressed as percentages of the control, which represented 100%.

### 2.5. Assessment of Interleukin 8 Concentration

The basal and zymosan-stimulated (10 μg·mL^−1^) production of IL-8 was measured from culture media collected after 24 h or 48 h of isorhamnetin treatment, using a commercial ELISA kit for human IL-8 determination (Invitrogen, ThermoFisher Scientific, Vantaa, FL, USA). The levels of IL-8 protein were measured in quadruplicates by quantitative, sandwich ELISA according to the manufacturer’s instructions. Plates were read at 450 nm of wavelength directly after the color reaction was stopped with acid. The absorbance values of the samples were determined using an ELISA reader (Multiscan FC, ThermoFisher Scientific, Vantaa, FL, USA). The IL-8 concentrations in the samples were quantified based on a calibration curve constructed from the calibrators included in the validated kit, with a new calibration curve constructed for each independent measurement.

### 2.6. Statistical Analysis

The data for statistical analysis were obtained from at least four independent experiments for each method. The GraphPad Prism tool (version 5 for Windows; GraphPad Software 5.03, La Jolla, CA, USA, www.graphpad.com (accessed on 13 August 2022)) was used to conduct the statistical analysis. Basic statistical characteristics were computed, and significant differences were determined using the one-way analysis of variance (ANOVA). The Shapiro–Wilk normality test was used to evaluate the normality of the variables. ANOVA was followed up with Dunnett’s test, which computes a confidence interval for the difference between two means by comparing each mean to a control mean. The differences were compared to assess statistical significance at the levels of *p* < 0.001 (***); *p* < 0.01 (**) and *p* < 0.05 (*).

## 3. Results

### 3.1. Effect of Isorhamnetin on Mitochondrial Activity of HT-29 Cells

**Figure 1:**
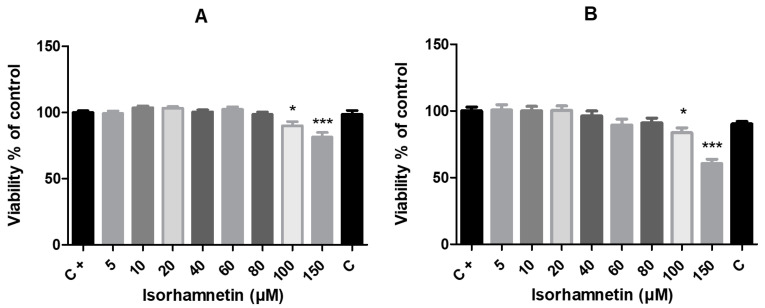
The impact of isorhamnetin on HT-29 cells’ mitochondrial activity after 24 h (**A**) and 48 h (**B**) of treatment. Each bar represents the mean value (±SEM) of the mitochondrial activity as a percentage of the control group (C+–DMSO group; isorhamnetin untreated cells), which represents 100%. The data are expressed as % of the control group. The data were collected from four (*n* = 4) separate experiments with cells of different passages. The levels of significance between the control and experimental groups were set as follows: *** (*p* < 0.001) and * (*p* < 0.05).

The in vitro effect of isorhamnetin on HT-29 colon cancer cells’ mitochondrial activity was determined by MTT assay. The cell viability demonstrated by the mitochondrial activity of exposed cells after 24 and 48 h of treatment was only significantly impacted by the tested amounts of isorhamnetin at the highest doses. As seen in Figure 1, for both culture times (24 h and 48 h), we observed significantly reduced values with the MTT test in the experimental groups of cells cultured in the presence of isorhamnetin at concentrations of 100 μM (*p* < 0.05) and 150 μM (*p* < 0.001) with respect to the control group.

### 3.2. Effect of Isorhamnetin on Metabolic Activity of HT-29 Cells

**Figure 2:**
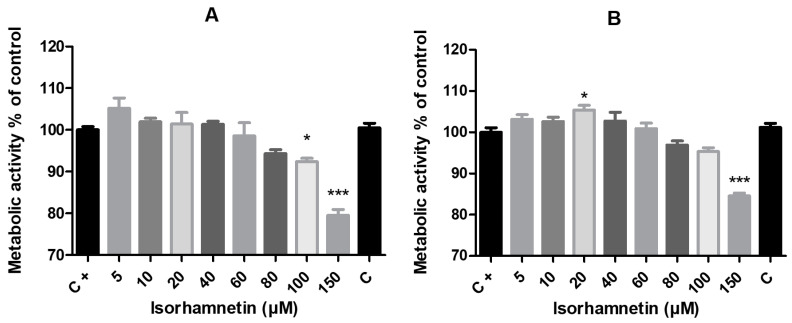
The impact of isorhamnetin on HT-29 cells’ metabolic activity after 24 h (**A**) and 48 h (**B**) of treatment. Each bar represents the mean value (±SEM) of the metabolic activity as a percentage of the control group (C+–DMSO group; isorhamnetin untreated cells), which represents 100%. The data are expressed as % of the control group. The data were collected from four (*n* = 4) separate experiments with cells of different passages. The levels of significance between the control and experimental groups were set as follows: *** (*p* < 0.001) and * (*p* < 0.05).

Our assessment of the metabolic activity, which reflects the rate of cell proliferation, using the alamarBlue test revealed that isorhamnetin treatment for 24 h led to a significant decrease in the values of samples cultured with the presence of 100 μM (*p* < 0.05) and 150 μM (*p* < 0.001). The experimental group with the highest isorhamnetin concentration (150 μM) showed significantly (*p* < 0.001) lower values after 48 h of exposure (Figure 2). However, during the 48 h of exposure to isorhamnetin, its stimulating effect manifested when the values of metabolic activity showed significant increases (*p* < 0.05) in cells treated with 20 μM when compared to cells of the control group (Figure 2).

### 3.3. Effect of Isorhamnetin on Membrane Integrity of HT-29 Cells

**Figure 3:**
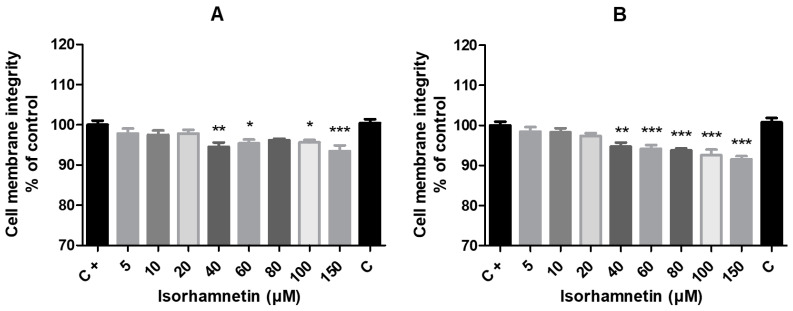
The impact of isorhamnetin on HT-29 cells’ membrane integrity after 24 h (**A**) and 48 h (**B**) of treatment. Each bar represents the mean value (±SEM) of the cell membrane integrity as a percentage of the control group (C+–DMSO group; isorhamnetin untreated cells), which represents 100%. The data are expressed as % of the control group. The data were collected from four (*n* = 4) separate experiments with cells of different passages. The levels of significance between the control and experimental groups were set as follows: *** (*p* < 0.001), ** (*p* < 0.01) and * (*p* < 0.05).

The effect of isorhamnetin on cell membrane integrity in HT-29 cells is presented in Figure 3. As compared to the previously monitored cell viability parameters, the effect of isorhamnetin on the observed parameter of exposed cells, in this case, was more pronounced, which was shown by altering the membrane integrity of cells even at lower isorhamnetin concentrations. A significant decline in the values of cells cultured with the tested compound for 24 h was noted in experimental group exposed to 40 μM (*p* < 0.01), 60 and 80 μM (*p* < 0.05) and 150 μM (*p* < 0.001) when compared to untreated cells. With respect to the 48 h treatment, isorhamnetin exhibited significantly reduced values of membrane integrity at concentrations of 40 μM (*p* < 0.01) and 60, 80, 100 and 150 μM (*p* < 0.001).

### 3.4. Effect of Isorhamnetin on Lysosomal Activity of HT-29 Cells

**Figure 4:**
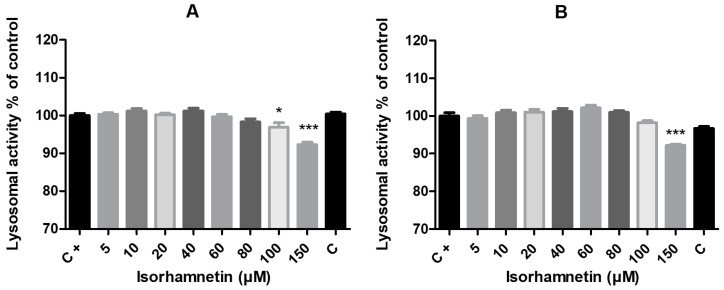
The impact of isorhamnetin on HT-29 cells’ lysosomal activity after 24 h (**A**) and 48 h (**B**) of treatment. Each bar represents the mean value (±SEM) of the lysosomal activity as a percentage of the control group (C+–DMSO group; isorhamnetin untreated cells), which represents 100%. The data are expressed as % of the control group. The data were collected from four (*n* = 4) separate experiments with cells of different passages. The levels of significance between the control and experimental groups were set as follows: *** (*p* < 0.001) and * (*p* < 0.05).

As shown by the neutral red test, the integrity of the lysosomes was affected only in the presence of the highest concentrations of isorhamnetin, when the administration of isorhamnetin resulted in the declining lysosomal activity of cells. In the case of the 24 h treatment, there was a significant change in the experimental group that received isorhamnetin at concentrations of 100 μM (*p* < 0.05) and 150 μM (*p* < 0.001), with the same result for 150 μM (*p* < 0.001) after 48 h of exposure (Figure 4).

### 3.5. Effect of Isorhamnetin on Intracellular Generation of Superoxide Radical in HT-29 Cells

**Figure 5:**
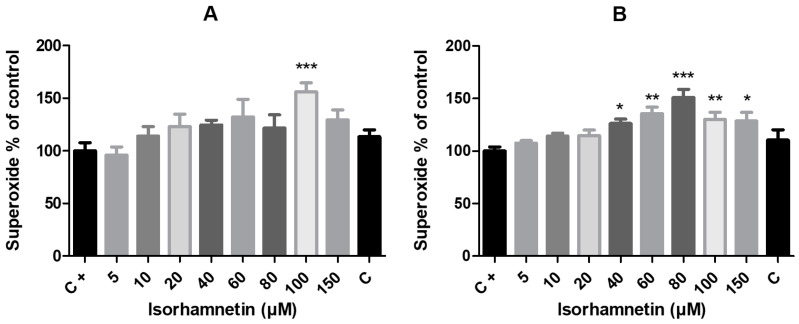
The impact of isorhamnetin on the intracellular production of superoxide radicals in HT-29 cells after 24 h (**A**) and 48 h (**B**) of treatment. Each bar represents the mean value (±SEM) of the superoxide concentration as a percentage of the control group (C+–DMSO group; isorhamnetin untreated cells), which represents 100%. The data are expressed as % of the control group. The data were collected from four (*n* = 4) separate experiments with cells of different passages. The levels of significance between the control and experimental groups were set as follows: *** (*p* < 0.001), ** (*p* < 0.01) and * (*p* < 0.05).

One of the main reactive oxygen species is intracellularly produced superoxide radicals, the high concentration of which is associated with the onset of oxidative stress in cells. According to the results of our study (Figure 5), isorhamnetin treatment, in a dose-dependent manner, led to increased concentrations of intracellular superoxide radicals, which indicate its prooxidative properties in HT-29 cells, particularly in higher doses. Despite the low values of the viability parameters in these samples, we observed a significant increase (*p* < 0.001) in the superoxide radical concentration in the experimental group treated with 100 μM of isorhamnetin after 24 h of exposure. After 48 h of exposure, there were more significant alterations in superoxide radical generation within the cells. Significantly higher concentrations of superoxide were observed in cells treated with isorhamnetin at 40 and 150 μM (*p* < 0.05), 60 and 100 μM (*p* < 0.01) and 80 μM, with the most significant (*p* < 0.001) effect compared to untreated cells of the control group.

### 3.6. Effect of Isorhamnetin on Interleukin 8 Production by HT-29 Cells

**Figure 6:**
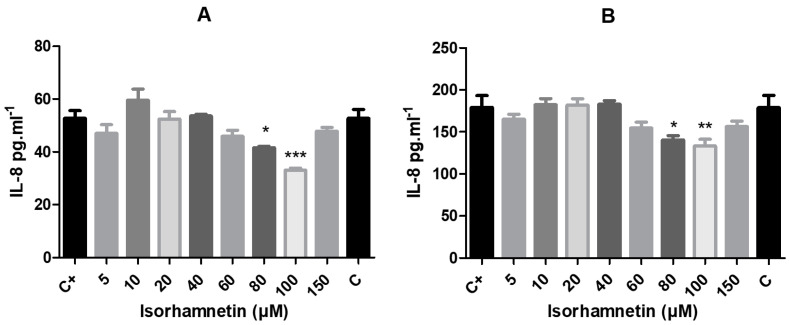
The impact of isorhamnetin on interleukin 8 production by HT-29 cells with 24 h (**A**) and 48 h (**B**) of treatment. Each bar represents the mean value (±SEM) of the IL-8 concentration (pg·mL^−1^) in the experimental groups compared to control group (C+–DMSO group; isorhamnetin untreated cells). The data were collected from four (*n* = 4) separate experiments with cells of different passages. The levels of significance between the control and experimental groups were set as follows: *** (*p* < 0.001), ** (*p* < 0.01) and * (*p* < 0.05).

Regarding the effect of isorhamnetin on interleukin 8 biosynthesis, its concentration was determined in basal conditions as well as after the stimulation of cells using zymosan. With the presence of isorhamnetin, 24 h of cultivation led to significant changes, with decreased production of IL-8 in HT-29 cells supplemented with isorhamnetin at concentrations of 80 μM (*p* < 0.05) and 100 μM (*p* < 0.001). The same doses of isorhamnetin led to a significant decline in the IL-8 concentration after 48 h of exposure (*p* < 0.05 in 80 μM; *p* < 0.01 in 100 μM; Figure 6).

**Figure 7:**
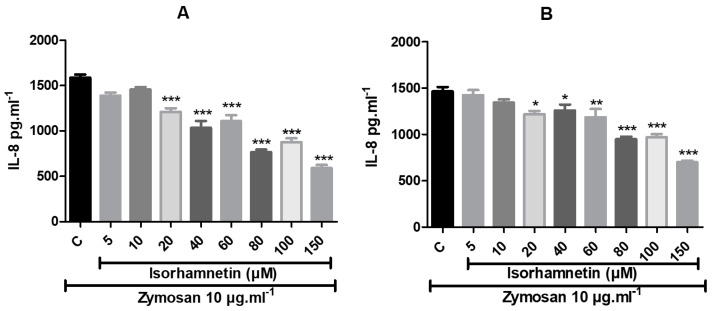
The impact of isorhamnetin on interleukin 8 production by zymosan-stimulated (10 μg·mL^−1^) HT-29 cells with 24 h (**A**) and 48 h (**B**) of treatment. Each bar represents the mean value (±SEM) of the IL-8 concentration (pg·mL^−1^) in the experimental groups compared to the control group (C–zymosan group). The data were collected from four (*n* = 4) separate experiments with cells of different passages. The levels of significance between the control and experimental groups were set as follows: *** (*p* < 0.001), ** (*p* < 0.01) and * (*p* < 0.05).

IL-8 biosynthesis after stimulation using zymosan (Figure 7) was significantly affected even at an isorhamnetin concentration of 20 μM, whereas IL-8 concentrations showed a decreasing trend with increasing concentrations of the tested substance. Exposure for 24 h revealed significantly reduced production of IL-8 in the experimental groups supplemented with isorhamnetin at doses of 20 μM, 40 μM, 60 μM, 80 μM, 100 μM and 150 μM (*p* < 0.001). After 48 h of treatment, released IL-8 concentrations were detected in culture media, with significantly decreased values in samples supplemented with isorhamnetin at concentrations of 20 μM, 40 μM (*p* < 0.05) and 60 μM (*p* < 0.01), as well as at 80 μM, 100 μM and 150 μM (*p* < 0.001) compared to the controls.

## 4. Discussion

According to a World Health Organization (WHO) report, tumor morbidity and mortality have still been rising gradually in recent years. Because their secondary metabolites are diverse and complex chemical components with high biological activity, natural compounds originating from plants have become a key source for medication research. Due to their low toxicity and wide range of targets and curative effects, natural compounds have received significant interest in the field of anti-cancer treatment [37]. Ethnopharmaceuticals rich in isorhamnetin have long been used as traditional herbal medicines for centuries for the treatment of cardiovascular diseases, chronic hepatitis, cancer, inflammation and other different pathological conditions in organisms [24]. Isorhamnetin has been shown in numerous experimental trials to have a variety of pharmacological actions, including antioxidant and anticancer properties. It has also been demonstrated that isorhamnetin has the ability to downregulate the expression of several inflammatory proteins, such as tumor necrosis factor-α, cyclooxygenase-2, prostaglandin E2, nuclear factor κB (NF-κB) and different interleukins [38,39]. The anticancer effect of isorhamnetin is attributed to the ability of this substance to activate apoptotic pathways in cancer cells by upregulating p53 and inducing the expression of the apoptotic factors B-cell lymphoma 2-associated X protein and caspase-2 [27,40].

Our results show that isorhamnetin is able to induce both dose- and time-dependent growth inhibitory effects in HT-29 cells. We found that isorhamnetin has a potent cytotoxic effect against colon cancer cells, especially in its highest tested concentrations after both cultivation periods (24 or 48 h), which was demonstrated by the inhibition of mitochondrial activity at concentrations of 100 and 150 μM with cultivation times of 24 h and 48 h. Similar results were observed in the case of the metabolic activity and lysosomal activity of the cells, while membrane integrity was already negatively affected in cells cultivated in the presence of isorhamnetin at a concentration of 40 μM, with a tendency of values to decline in cells with increasing isorhamnetin concentrations after 48 h of exposure. In the context of colon cancer, our results are in agreement with in vitro study that pointed to a cytotoxic effect of isorhamnetin on Caco2 human colon cancer cells [24]. This study evaluated the effect of isorhamnetin glycosides in the form of Opuntia Ficus-indica extract (77.32 ± 3.8 mg isorhamnetin/g), which showed cytotoxic and apoptic effects, with increased activity of caspase 3/7 in exposed cells. Jaramillo et al. [41] studied another type of human colon carcinoma cells (HCT-116) and interpreted antiproliferative, apoptotic, necrotic and cell cycle modulatory effects suggesting that isorhamnetin may have clinically significant capabilities. With an IC50 of 72 μM after 48 h of incubation, as estimated by MTT assay, isorhamnetin also exerted a stimulatory effect on apoptosis and necrosis with the increased number of cells in the G2/M phase. These findings are consistent with the notion that G2/M cell cycle checkpoints may be a conserved target for flavonoids in cancer cells, leading to apoptotic and necrotic death, and that isorhamnetin may mediate the inhibition of human colon cancer cell growth and proliferation through the disruption of cell cycle progression [41]. Isorhamnetin was previously also shown to reduce the proliferation of esophageal tumor cell line Eca-109 [42], human hepatocellular carcinoma BEL-7402 cells [43] and murine Lewis lung cancer cells [27]. Flavonoids’ ability to inhibit the growth and proliferation of several cancer cell types has been linked to a number of different mechanisms of action, including the expression of tumor suppressor genes and oncogene control; the induction of cell cycle arrest and apoptosis involving the p53, Bcl-2 and families of caspase activation; and the inhibition of signal transduction pathways such as Nrf, NF-κB, AP-1, Wnt/β-catenin, MAPK and growth factors [44].

Since ROS were found to play a key role in the activation of pro-apoptotic death signals in cancer cells, the effect of isorhamnetin on intracellular superoxide accumulation in HT-29 cells was assessed in this study. The results obtained from NBT test showed that samples supplemented with 100 μM isorhamnetin, despite significantly reduced cell viability parameters, had significantly increased concentrations of superoxide radicals after 24 h of exposure in relation to the control. A similar situation occurred after 48 h of treatment, when superoxide production began to significantly rise at a concentration of 40 μM and continued rising with higher concentrations of isorhamnetin.

Currently, it is well established that flavonoids have a dual effect on ROS homeostasis in cells—in normal circumstances, they function as antioxidants, but when cancer cells are present, they become potent pro-oxidants triggering the apoptotic pathways and downregulating pro-inflammatory signaling pathways [45]. Due to their capacity to stabilize free radicals in the presence of phenolic hydroxyl groups, flavonoids have the ability to directly scavenge ROS and chelate metal ions. Indirect flavonoid antioxidant effects are related to the activation of antioxidant enzymes, the suppression of pro-oxidant enzymes, and stimulating the production of antioxidant enzymes and phase II detoxification enzymes. Depending on the particular cancer cell type, both pro-oxidant and antioxidant activities seem to be implicated in the anticancer actions of flavonoids [46]. A large body of evidence has shown that most anticancer agents enhance apoptosis for the elimination of cancer cells through pro-oxidant properties, such as increasing ROS accumulation or degrading cellular antioxidant systems. Elevated concentrations of free radicals, such as reactive oxygen species, within cells are considered to be one of the effective mechanisms for destroying cancer cells through the activation of intrinsic pathways [47]. As is consistent with a previous study on breast cancer cells [21] and hepatocarcinoma cells [48], our outcomes show that isorhamnetin treatment significantly stimulated the generation of ROS, specifically intracellular superoxide radicals. The study of Park et al. [34] also demonstrated stimulated production of intracellular reactive oxygen species by isorhamnetin when the interruption of ROS generation using a ROS scavenger led to an escape from isorhamnetin-mediated G2/M arrest and apoptosis in human bladder cancer cells.

The study of the molecular and cellular mechanisms of cancer has shown a strong regulation of carcinogenesis induction and progression by the inflammation processes that manage the microenvironments of tumors. Many experiments have stated that cytokines are not only released by the immune cells occurring in the tumor microenvironment, but an autocrine source of some inflammatory cytokines (IL-1, IL-4, IL-6 and IL-8) was also confirmed in a large spectrum of solid tumors. Additionally, cancer cells also express related receptors to be used to escape from the immune response [49]. The inhibition of angiogenesis, which is the development of new blood vessels from the pre-existing network, is thought to be an effective approach by which to suppress a wide variety of tumor growth. A well-known cytokine that promotes inflammation and serves as a key chemoattractant for leukocytes is IL-8. However, as must be mentioned in the context of carcinogenesis, it has been demonstrated that IL-8 contributes to the growth, migration and metastasis of colon cancer cells, both in vitro and in vivo, by acting as a mitogenic, migratory and angiogenic factor [8]. Moreover, the existence of this mechanism is supported by the fact that elevated levels of IL-8 in serum and cancer tissue have been observed in colorectal patients, and it has been confirmed that IL-8 expression in tumor tissue is significantly correlated with tumor size, depth of infiltration and metastasis in the liver, as well as with the stage of the tumor [50].

In order to determine whether isorhamnetin could alter interleukin 8 biosynthesis, in this study, we activated a human colon cancer cell line to an inflammatory state using zymosan and measured the concentration levels of IL-8. The level of basal IL-8 production at without cell stimulation was also assessed. Our results revealed that the basal production of IL-8 was effectively inhibited after isorhamnetin treatment with 80 μM and 100 μM and 24 and 48 h of cultivation. In the case of stimulation of HT-29 cells using zymosan, IL-8 expression was altered in a positive manner in the experimental groups, even with the presence of lower concentrations of isorhamnetin (20, 40, 60, 80, 100 and 150 μM) after 24 and 48 h of cultivation.

The findings from an earlier study, which show that isorhamnetin can prevent BV2 microglia cells from undergoing LPS-induced inflammatory signaling, support our findings. It has been proven that isorhamnetin works by suppressing the expression of TNF- and IL-1 in these cells to lower their release [51]. The results of an experiment using a model of gingival fibroblasts showed that isorhamnetin could considerably reduce the generation of NO and PGE2 when there was no cytotoxicity present, which was also accompanied by a decrease in iNOS and COX-2 expression [52]. These studies suggest that isorhamnetin may improve the inflammatory response by inhibiting the expression of genes that regulate the production of pro-inflammatory factors.

## 5. Conclusions

Based on our findings, isorhamnetin appears to have an anti-proliferative effect on human colon cancer cells, which is associated with its pro-oxidant activity. The potential anticancer activity of isorhamnetin seems to also be mediated by the effective reduction in interleukin 8 production by cancer cells, whose elevated level is linked to cancer progression and metastasis through its functions as a factor that triggers cell division or accelerates mitosis, migration and angiogenesis. Modulation of this process of interleukin biosynthesis could, therefore, represent another mechanism that we could consider the potential anticancer activity of isorhamnetin. Therefore, these results provide evidence for the anti-cancer activity of isorhamnetin; however, more detailed research is needed to clarify the mechanism of action of isorhamnetin in colorectal cancer cells in order to objectively evaluate the therapeutic properties and effectiveness of this bioactive molecule for the prevention and therapy of colorectal cancer.

## Data Availability

All data are provided within the manuscript.
